# A Case of Long-term Survival after Curative Resection for Synchronous Solitary Adrenal Metastasis from Rectal Cancer

**DOI:** 10.12669/pjms.301.4341

**Published:** 2014

**Authors:** Linlin Chen, Da Wang, Weifang Mao, Xuefeng Huang, Chao He

**Affiliations:** 1 Linlin Chen, MD, Department of Colorectal Surgery, Sir Run Run Shaw Hospital, College of Medicine, Zhejiang University, Hangzhou, Zhejiang, 310016, China.; 2 Da Wang, MD, Department of Colorectal Surgery, Sir Run Run Shaw Hospital, College of Medicine, Zhejiang University, Hangzhou, Zhejiang, 310016, China.; 3 Weifang Mao, MD, Department of Colorectal Surgery, Sir Run Run Shaw Hospital, College of Medicine, Zhejiang University, Hangzhou, Zhejiang, 310016, China.; 4 Xuefeng Huang, MD, Department of Colorectal Surgery, Sir Run Run Shaw Hospital, College of Medicine, Zhejiang University, Hangzhou, Zhejiang, 310016, China.; 5 Chao He, Professor, Department of Colorectal Surgery, Sir Run Run Shaw Hospital, College of Medicine, Zhejiang University, Hangzhou, Zhejiang, 310016, China.

**Keywords:** Adrenalectomy, Long-term survival, Rectal cancer, Solitary adrenal metastasis

## Abstract

Clinically curable adrenal metastasis is rare. We report a case of synchronous solitary adrenal metastasis from rectal cancer in a 51-year-old man who underwent curative resection. A right adrenal mass was found by ultrasonography during his routine physical examination and this was confirmed by computed tomography (CT). His serum carcinoembryonic antigen (CEA) level was found elevated, and colonoscopy revealed a rectal tumor located 10cm from anal verge. A simultaneous laparoscopic right adrenalectomy and anterior resection for rectal carcinoma was performed. Histopathological examination revealed well-differentiated rectal adenocarcinoma with adrenal metastasis. The patient is still alive and free from disease 6 years after the surgery. A review in the literature showed that synchronous solitary adrenal metastasis from colorectal carcinoma is very rare. Surgical resection and for selected patients, laparoscopic procedure may provide survival benefit and potential surgical cure for a solitary metastasis.

## INTRODUCTION

Distant metastases to organs such as liver and lung are common in colorectal cancer patients. Approximately 20 percent of patients have liver metastatic disease at the time of presentation.^1^ Based on autopsy reports, adrenal gland metastasis is also not rare.^2^ Generally, adrenal metastasis is considered to be a widespread disease and with poor prognosis. Solitary and clinically curable adrenal metastasis from colorectal carcinoma is very rare.^3^ Here, we present a long-term survival case with rectal cancer and a synchronous solitary adrenal metastasis who underwent radical surgical resection.

## CASE REPORT

**Fig.1:**
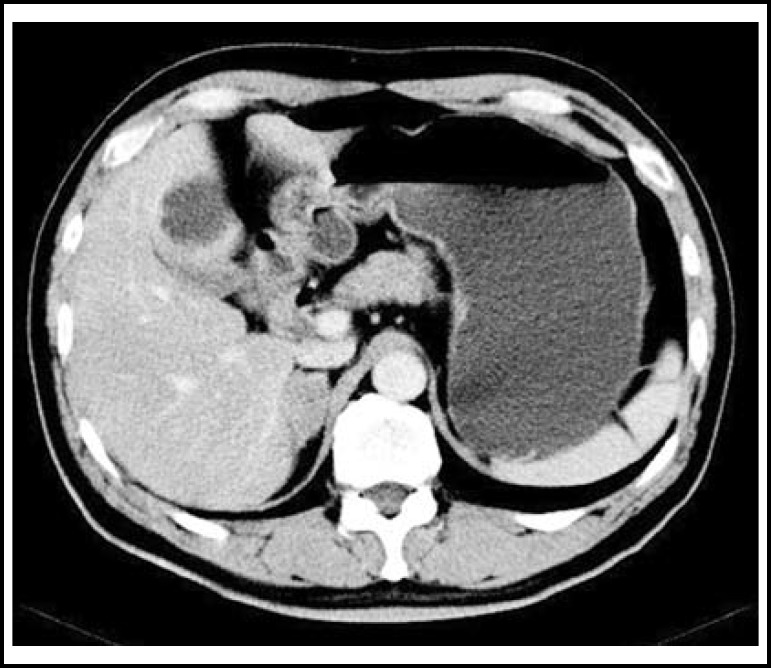
Enhanced CT scan demonstrating a right adrenal mass of low density

The patient was a 51-year-old man. A right adrenal mass was found by ultrasonography during his routine physical examination in December 2006. Computed tomography (CT) scan confirmed a moderately enhanced mass in the region of the right adrenal gland, which was 3.3×2.0 cm in size (Fig.1). Furthermore, his serum carcinoembryonic antigen (CEA) level was found elevated at 72.2ng/ml. Gastroscopy and colonoscopy were therefore performed. Colonoscopy revealed a rectal tumor of the ulcerating type located 10cm from anal verge and biopsy specimens confirmed it to be an adenocarcinoma. His chest X-ray and liver CT scan seemed normal. He was subjected to a simultaneous laparoscopic right adrenalectomy and anterior resection for rectal carcinoma on January 5, 2007. No frozen section was done during the procedure. Neither peritoneal seeding nor liver metastasis was observed during intraoperative exploration.

**Fig.2:**
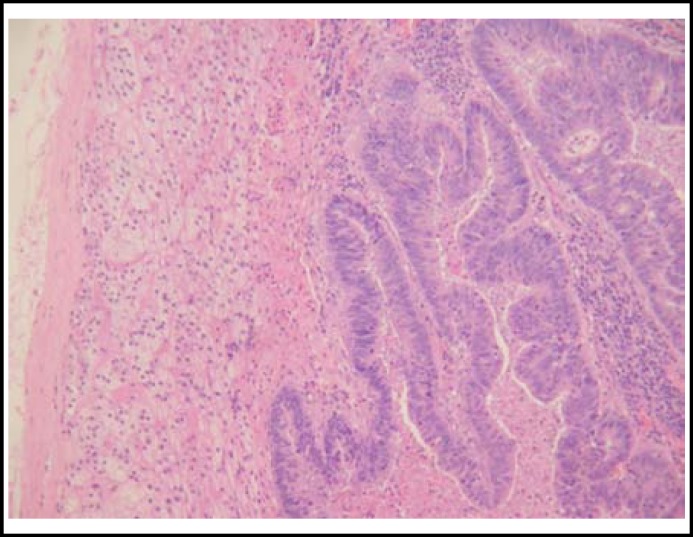
Microscopic appearance of the adrenal tumor showing a well-differentiated adenocarcinoma, similar to the primary rectal carcinoma (H&E ×100).

Postoperative pathological examinations showed that the rectal tumor, which measured 4.0×3.0 cm, was a well-differentiated adenocarcinoma that infiltrating to the adventitia layer, with metastasis to two of three dissected lymph nodes. The pathological findings of the right adrenal tumor revealed infiltrating adenocarcinoma within the normal adrenal gland, compatible with metastasis from the rectal carcinoma (Fig.2).

The patient’s postoperative course was good; he did not develop adrenal insufficiency and was discharged 14 days after surgery. His serum CEA level normalized to 4.5ng/ml one month postoperatively. Totally, he received 12 cycles of adjuvant chemotherapy with FOLFOX4 schedule. At last follow-up, in January 2013, he showed no evidence of recurrence or metastasis with a normal CT scan and serum CEA, 6 years after resection of his rectal cancer and synchronous solitary adrenal metastasis.

## DISCUSSION

The incidence of adrenal metastasis from colorectal carcinoma ranges from 0.15% to 17.4% with a mean percentage of 16%.^4^ Metastatic adrenal disease is usually part of a disease disseminated to several organs and has limited survival. Solitary adrenal disease is infrequent and always in a metachronous setting. Some of the reported cases include patients who had other distant metastases before the adrenal metastasis; strictly speaking, such patients might not be considered to have a solitary metastasis. By performing a Medline literature search, we were able to identify 16 previous reported cases of resection of solitary adrenal metastasis from colorectal carcinoma (Table-I). Only 6 of these 17 cases, including ours, the metastasis was synchronous.

Since 15 of 17 cases were male patients, solitary adrenal metastasis seems more frequently in male; with an average age over 61 (range 43-74). The primary carcinomas were mostly in rectum (10 cases), others were in sigmoid colon (3 cases), ascending colon (2 cases), descending colon (1 case) and cecum (1 case).

**Table-I:** Total 17 cases of solitary adrenal metastasis from colorectal carcinoma

*Author, year*	*Patient data*			*Colorectal carcinoma*		*Adrenal metastasis*		*Recurrence*		*Outcome*
	*No.*	*Age*	*Gender*	*Location*	*Duke’s*	*Interval*	*Size(cm)*	*Organ*	*Interval*	
Matsui, 1985	1	71	M	Rectum	B	2y	10×6.5×6.5	None	-	Alive at 8m
Watatani, 1993	2	52	M	Rectum	D	Syn	10.5×6×5	Liver	6m	Died at2y9m
Watatani, 1993	3	66	M	Cecum	C	1y 6m	9×8	None	-	Alive at 1y
Fujii, 1994	4	57	M	Ascending	B	3y 1m	6×4×3	None	-	Alive at 9y
Mizutani, 1995	5	58	M	Rectum	B	1y 2m	4.0×3.5×2.0	Lung	4m	Died at 2y 7m
Kamasako, 1995	6	71	F	Sigmoid	D	Syn	6.5×5.0×3.5	Liver	8m	Alive at 11m
Ozawa, 1996	7	46	M	Descending	C	1y 2m	10×9.1×5.7	None	-	Alive at 1y 2m
Shoji, 2006	8	73	M	Sigmoid	B	4m	5.3×3.3×2.4	Liver	12m	Died at 3y 2m
Kita, 2006	9	45	M	Rectum	D	Syn	?	None	-	Alive at 1y
Mourra, 2008	10	56	M	Rectum	D	Syn	1.8	None	-	Alive at 1y 10m
Mourra, 2008	11	61	M	Rectum	B	3y	3.0	Lung	?	Died at 2y
Mourra, 2008	12	66	M	Rectum	A	5y	15	Liver	?	Died at 4y
Bonfill, 2009	13	61	F	Rectum	D	Syn	6	None	-	Alive at 5y
Pascual, 2010	14	65	M	Rectum	?	9m	6.8	Bilateral	8m	Alive at 2y 10m
Thrumurthy, 2011	15	68	M	Sigmoid	B	1y	6×5×2	Bilateral	7m	Alive at 8y
Capaldi,2011	16	74	M	Ascending	B	3y 8m	4×5	None	-	Alive at 10m
Our case	17	51	M	Rectum	D	Syn	3.3×2.0	None	-	Alive at 6y

M: male; F: female; Syn: synchronous; y: years; m: months; ?: unknown (not described).

In all cases, patients with metastatic adrenal tumor were asymptomatic, without pain or adrenal insufficiency. All metastases were diagnosed by CT scan or elevation in CEA levels synchronously or in follow-up surveillance. Serum CEA level is a useful indicator for the presence of adrenal metastases. In most of the cases, including ours, CEA levels were elevated at the time of adrenal metastasis and decreased considerably after adrenalectomy. Only in one case (case 16), CEA level remained within the normal range. CT scan is a cost-effective means of identifying adrenal metastases. The findings of CT in our patient disclosed the low density of the adrenal gland, in accordance with reports by other authors.^5^^,^^6^ However, it does not provide any distinction between primary lesions and secondary lesions. Magnetic resonance imaging (MRI) is reported effective for distinguishing adenomas, carcinomas, and metastases in case of adrenal incidentaloma.^6^ Recently, A few reports and studies in the literature have suggested that fluorine 18-fluoro-deoxy-glucose positron emission tomography (18F-FDG PET-CT) is useful to differentiate benign from malignant adrenal lesions, with sensitivity ranging from 93 to 100%, specificity ranging from 80 to 100% and accuracy ranging from 92 to 100%**.**^7^ Although PET-CT was not performed preoperatively in our patient, we think it is useful to exclude metastatic disease elsewhere prior to a potentially curative adrenal resection. Candel et al.^8^ emphasized that fine-needle aspiration biopsy of adrenal masses was helpful for making an accurate diagnosis. However, in our opinion, this risky technique in characterizing adrenal masses, may eventually be assumed by the less invasive and more accurate PET-CT.

It is thought that there are several routes by which colorectal cancer can metastasize to the adrenal glands, including systemic venous, portal venous, arterial and lymphatic routes. The 4 latent liver metastases (case 2, 6, 8, 12) and 2 latent lung metastases (case 5, 11) 4-12 months after adrenalectomy might suggest that the route from primary lesion via liver or lungs to systemic venous circulation and silent liver or lung metastases had already occurred at the time of detection of the adrenal metastases. In contrast, there were 3 patients, including ours (2 synchronous, 1 metachronous, case 4, 13, 17) had no other metastases detected during the long-term (>5 years) follow-up.

The follow-up period of the 17 patients after resection of adrenal metastases ranged from 8 months to 9 years. Five patients died of recurrence with extensive metastases to multiple organs 24-48 months (median 33 months) after adrenalectomy. One patient showed liver recurrence 8 months after adrenalectomy, but was still alive at the time of report. Two patients presented metachronous bilateral adrenal metastases, after operations, they were alive without metastatic and recurrent disease at the time of report. Nine patients were alive without signs of recurrence 8 months-9 years after adrenalectomy (median follow-up period of 14 months). In the 6 synchronous adrenal metastases patients, 4, including ours, were alive without signs of recurrence at the time of report. These data suggest that resection of solitary adrenal metastasis from colorectal carcinoma may improve patients’ chances of survival. Our long-term survival case also suggests that synchronous solitary adrenal metastasis may respond well to radical surgical resection, chemotherapy and vigilant follow-up.

With technological advances, laparoscopic adrenalectomy is now considered as the gold-standard approach for small benign secreting tumors, but it remains a matter of debate for large and potentially malignant adrenal tumors and adrenal metastases.^9^ Recently, several reports have described long-term survival after laparoscopic adrenalectomy for solitary adrenal metastasis^.^^10^^,^^11^ However, no prospective randomized studies have compared open versus laparoscopic resection of solitary adrenal metastasis, because it is difficult to recruit patients. In our case, since the adrenal mass was relatively small (3.3×2.0 cm) and no signs of invasion of the surrounding organs or lymph nodes, we performed laparoscopic approach. It suggests that laparoscopic adrenalectomy for small solitary metastasis is a feasible procedure.

In conclusion, synchronous solitary adrenal metastasis from colorectal carcinoma is very rare. On the basis of our case and international literature, we believe that surgical resection and for selected patients, laparoscopic procedure may provide survival benefit and potential surgical cure for a solitary metastasis.
